# A double-edged sword: unusual multiple severe infections with pralsetinib: a case report and literature review

**DOI:** 10.3389/fmed.2024.1402902

**Published:** 2024-07-30

**Authors:** François Poumeaud, Marion Jaffrelot, Carlos Gomez-Roca, Iphigénie Korakis, Giulia Leonardi, Marine Joly, Julien Mazières, Rosine Guimbaud, Nadim Fares, Emily Alouani

**Affiliations:** ^1^Department of Medical Oncology, Oncopole Claudius Regaud, Toulouse, France; ^2^Department of Digestive Oncology, Centre Hospitalier Universitaire de Toulouse, Toulouse, France; ^3^Clinical Research Unit, Department of Medical Oncology, Oncopole Claudius Regaud, Toulouse, France; ^4^Department of Infectiology, Centre Hospitalier Universitaire de Toulouse, Toulouse, France; ^5^Department of Thoracic Oncology, Centre Hospitalier Universitaire de Toulouse, Toulouse, France

**Keywords:** pralsetinib, RET inhibitor, infection, JAK/STAT, case report

## Abstract

Selective rearranged during transfection (RET) tyrosine kinase inhibitor, pralsetinib, demonstrated clinical efficacy and was well tolerated in lung and thyroid cancers with *RET* gene mutations or fusions in clinical trials. While the latter focused on the risk of pneumonitis, there is a lack of data regarding other types of infectious risks associated with pralsetinib. Herein, we report the case of a 53-year-old patient with a *CCDC6-RET* fusion neuroendocrine tumor, who achieved a partial response with pralsetinib as the fifth-line therapy. Of particular note, during pralsetinib therapy, the clinical course was complicated by five severe infectious events, namely, two oxygen-requiring pneumonias, two distinct spondylodiscitis, and one pneumocystis. Our study highlights the increased risk of any type of opportunistic infectious event with pralsetinib, but not selpercatinib, which is probably caused by off-target JAK1/2 inhibition.

## Introduction

The aberrant activation of rearranged during transfection (*RET*) proto-oncogene is a critical factor of oncogenesis in diverse solid tumors. Germline *RET* mutations cause multiple endocrine neoplasia type II, leading to the occurrence of medullary thyroid cancers (MTC) and neuroendocrine tumors such as pheochromocytomas (1). Selective RET tyrosine kinase inhibitors demonstrated striking clinical efficacy in lung and thyroid cancers with *RET* gene mutations or fusion with an overall response rate as high as 70% among treatment-naive patients (2). This theragnostic impact has led to phase I/II basket trials in patients with *RET*-altered pan-cancers, where RET inhibitors showed significant clinical efficacy, with objective response rate (ORR) varying from 43.9 to 50% (3, 4). There are currently two inhibitors, namely, pralsetinib (BLU-667 or X581238) and selpercatinib (LOXO 292), that are FDA and EMA approved based on ARROW trials and the LIBRETTO-001, for only *RET*-altered non-small cell lung cancer (NSCLC) and thyroid cancer (5) and locally advanced or metastatic *RET* fusion-positive solid tumors, respectively. The advent of tumor molecular boards and shifting treatment paradigms toward biomarker-guided tumor-agnostic approaches for the current cancer management will extend the use of such treatments. Indeed, *RET* testing is currently used in routine in next-generation sequencing (NGS) panels, which identified 2% of *RET* alterations in various types of cancer, including digestive, breast, ovarian, and head-and-neck cancers (6). It is therefore important to precisely establish the safety profile of these new emerging treatments. There is mounting clinical evidence of severe lung infections associated with pralsetinib, but there is little evidence of other infectious events. Here, we report the first case of multiple severe infections in one patient treated with pralsetinib for *RET*-rearranged metastatic pancreatic neuroendocrine tumor and provide a narrative review to discuss available clinical evidence on pralsetinib-related infectious toxicities as compared to selpercatinib.

## Case report

Herein, we report the case of a 53-year-old man, followed since early 2020 for a grade III, well-differentiated, pancreatic neuroendocrine tumor with synchronous bone, nodal, liver, and lung metastases. His past medical history mainly includes hereditary polyneuropathy (Charcot–Marie–Tooth disease) without clinical invalidity. The patient successively received standard systemic treatments such as gemcitabine-oxaliplatin, capecitabine-temozolomide, carboplatin-etoposide (VP-16), and sunitinib from January 2020 to June 2022. The objective response rate with previous systemic therapy ranged from partial response to stable disease. Oncologic treatments were discontinued for progressive diseases and there was no arrest due to toxicity. Agnostic tumoral NGS (TSO500 panel) performed after progression under sunitinib treatments identified a *CCDC6-RET* fusion. The patient was enrolled in a tumor-agnostic platform clinical trial and received pralsetinib at a dose of 400 mg/day from 27^th^ September 2022 as the fifth-line treatment. Notably, this patient had neither experienced any infectious events or complications during his oncologic history before pralsetinib initiation nor presented lymphopenia or neutropenia, which would suggest some types of immunosuppression. The patient had no corticosteroid treatment at pralsetinib initiation.

**Figure 1:**
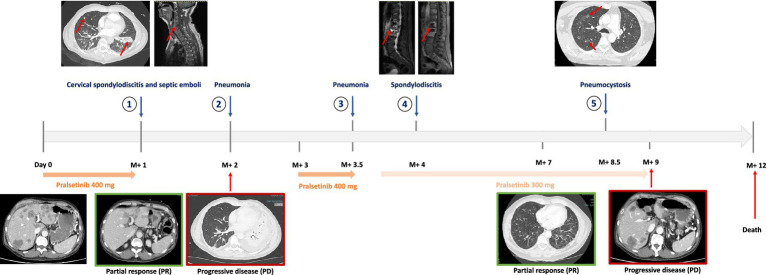
Timeline of infections and therapeutic outcomes. The patient experienced five infectious events under the pralsetinib therapy with oncologic progression during pralsetinib discontinuation. The patient experienced two infectious events at a reduced dose of 300 mg. M+, months since pralsetinib initiation.

One month after pralsetinib initiation (5 November 2022), he developed symptomatic *Pseudomonas aeruginosa* bacteremia (wild-type phenotype) complicated with lung septic embolisms and concomitant grade III cervical spondylodiscitis involving the 5th and 6th cervical vertebrae and para-vertebral C2 to C5 abscess. Pralsetinib was immediately discontinued, and the patient was successfully treated with piperacillin-tazobactam and ciprofloxacin antibiotic therapy for 14 days, followed by ciprofloxacin monotherapy for 4 more weeks. Transthoracic echocardiography (TTE) excluded infective endocarditis. Notably, the computational tomodensitometry (CT) performed to diagnose septic embolisms also reported a tumor partial response (PR), according to RECIST (Figure 1). After 3 weeks of ciprofloxacin monotherapy and resuming without pralsetinib (as of 5th December 2022), the patient was diagnosed with a second infectious episode of grade II pneumonia, for which he received 7 days of amoxicillin-clavulanic acid. After 1 month of pralsetinib discontinuation due to these two infectious events, a full CT-scan re-evaluation was performed, which revealed new secondary lesions with pleural effusion, peritoneal carcinosis, and lytic bone lesions. Pralsetinib was then resumed on 23^rd^ December 2022 at a standard dose of 400 mg per day, with rapid clinical benefits. After 3 weeks of reintroduction (12^th^ January 2023), the patient experienced a third infectious episode with grade III pneumonia requiring hospitalization due to grade II neutropenia. This was successfully treated with 7 days of piperacillin-tazobactam. After 1 week of reintroduction, the patient presented with febrile back pain and a 4/5 motor deficit in the right leg. Magnetic resonance imaging (MRI) identified typical spondylodiscitis involving 1^st^ to 3^rd^ lumbar vertebrae. Considering the recurrent infectious events, an extensive infectious immune workout was performed. The vertebral biopsy identified the presence of *P. aeruginosa*, and the histology did not show the presence of bone tuberculosis or tumor progression. Repeated blood culture (bacterial and fungal) was performed and remained negative, and transesophageal echocardiography (TEO) was normal. Grade I lymphopenia was identified without concomitant neutropenia. Lymphocyte immunophenotyping did not reveal any imbalance between CD4 and CD8 sub-populations, B lymphopenia, and natural killer (NK) deficiency. However, plasmatic protein electrophoresis identified deep hypogammaglobulinemia with specific IgG deficiency without a decrease in IgA and IgM. Broad-spectrum serology, including human immunodeficiency virus (HIV) and hepatitis B and C, was negative. This fourth infectious episode was considered as a grade III lumbar spondylodiscitis, which required 2-week hospitalization, and was successfully treated with tobramycin and ciprofloxacin for 48 h followed by a high dose of piperacilline-tazobactam (16 g per day) and ciprofloxacin for 2 weeks, as well as monotherapy with ciprofloxacin for another 2 months. Pralsetinib was continued at 300 mg, and the patient experienced a new partial response in May 2023. Later, on 15th June 2023, in the context of persistent lymphopenia and febrile hypoxemia, a thoracic CT scan was performed and showed disseminated, poorly delimitated pulmonary lesions, which were first interpreted as disease progression. However, pulmonary secretion was positive for *Pneumocystis jirovecii*. The final diagnosis was a symptomatic pneumocystis, which was treated with high-dose trimethoprim/sulfamethoxazole. Pralsetinib was discontinued after the fifth episode. Finally, the patient presented with liver progression on 30 June 2023, and pralsetinib was definitively discontinued (Figure 1). The patient died in the palliative care unit in September 2023, 3 years after diagnosis, including 9 months of oncologic clinical treatment with pralsetinib.

## Discussion

To the best of our knowledge, we report here the first case of recurrent severe infections characterized by “on/off” episodes under pralsetinib therapy. While clinical trials and most case reports (7–10) focused on the risk of pneumonitis in patients under pralsetinib therapy [grade 3 pneumonia in 3% (2) to 10.7% (11) of patients], our case report and literature review demonstrate the risk of various types of opportunistic infectious events. In some cases, patients treated with pralsetinib presented hematogenous tuberculosis, pulmonary cryptococcosis, one grade V typhlitis, and one grade V sepsis, as shown in Table 1 (7–9, 12, 14). Moreover, it is important to note that these infections can be severe, with 4.1% of fatal events reported by EMA. According to these data, infectious events may be a significant safety issue for patients receiving pralsetinib. Moreover, no risk factor for infection under the pralsetinib therapy has been identified so far.

**Table 1:** Literature review of opportunistic infections reported with pralsetinib.

Number	Sex	Age	Comorbidities	Cancer	RET alteration	Posology (mg)	Leucopenia at time of infection	Time to first infection (mo)	Type of infection	Best ORR	Drug management	Reference
1	Male	81	Type II diabetes mellitus Coronary heart disease	NSCLC	KIF5B-RET fusion	300	NA	4.5	Pulmonary cryptococcosis	SD	Restarted at 200 mg.	An et al. (8)
						200	No	8	S. Aureus pneumonia		NA	
2	Male	55	NA	NSCLC	7 KIF5B-RET fusion 1 KIAA1468-RET fusion	NA	NA	2	Pneumospora Yerbii. Aspergillosis	5 PR 3 SD	NA	Gao et al. (7)
3	Female	47	NA	NSCLC		NA	NA	2	Pneumospora Yerbii. HHV		NA	
4	Female	44	NA	NSCLC		NA	NA	7	Pulmonary pneumocystosis		NA	
5	Male	81	NA	NSCLC		NA	NA	5	Pulmonary cryptococcosis S. Aureus pneumonia		NA	
6	Female	57	NA	NSCLC		NA	NA	2.5	Multiple germs pneumonia Aspergillosis		NA	
7	Female	57	NA	NSCLC		NA	NA	2.5	Pneumospora yerbii.		NA	
8	Female	54	NA	NSCLC		NA	NA	2	CMV, EBV		NA	
9	Female	64	NA	NSCLC		NA	NA	2.5	CMV		NA	
10	Female	57	No	NSCLC	KIF5B-RET fusion	300	No	1.5	Hematogenous tuberculosis	Clinical response	Restarted at 300 mg after 2 months of antibiotics and with.	Lee et al. (12)
11	Female	51	No	NSCLC	CCDC6-RET fusion	300	No	2	Hematogenous Tuberculosis	PR	Restarted at 300 mg after 2 weeks of antibiotics.	
						300	NA	6	Herpes zoster		Resumed at 300 mg	
12	Female	58	Non-bacterial thrombotic endocarditis	NSCLC	NA	NA	Grade I neutropenia	NA	*Herbaspirillum* pneumonitis	NA	NA	Dhital et al. (10)
13	Male	62	No	Papillary thyroid cancer	NA	NA	NA	9	Endobronchial tuberculosis	PR	NA	Bolourchi et al. (13)
14	Female	63	Diabetes mellitus	NSCLC	KIF5B-RET and ATF6-RET fusions	300	No	4	Pneumonitis		Restarted at 200 mg	Chen et al. (9)
						200	Lymphopenia (CD4 and CD8)	4.5	Hematogenous tuberculosis	PR	Restarted at 200 mg after 1 month of antibiotics	
15	NA	NA	NA	NSCLC	NA	NA	NA	NA	Grade V Typhlitis	NA	Patient deceased, no reintroduction	Passaro et al. (14)
16	NA	NA	NA	NSCLC	NA	NA	NA	NA	Grade V sepsis	NA	Patient deceased, no reintroduction	
Our Case	Male	53	No	NET	CCDC6-RET fusion	400	No	1	Spondylodiscitis		No reintroduction	
						Withdraw	No	2	Pneumonia		Restarted after 1 month, at 400 mg
						400	Grade II pneumonia	3.5	Pneumonia	PR	Reintroduction at 300 mg
						300	Grade I Lymphopenia	4	Spondylodiscitis		Maintained at 300 mg
						300	No	8.5	Pneumocystis	–	Stopped for progression 2 weeks after

ORR, objective response rate; NA, non-available; NSCLC, non-small cell lung cancer; SD, stable disease; HHV, herpes human virus; PR, partial response; CMV, cytomegalovirus; EBV, Epstein–Barr virus; NET, neuroendocrine tumor; PD, progressive disease.

Another concern is the possible uncertainty between tumor progression and pralsetinib-associated infections. A retrospective study analyzed the occurrence of invasive pulmonary mycosis in patients with NSCLC, and among the 13 patients diagnosed with pulmonary mycosis, 62% of patients were initially misdiagnosed as metastatic or recurrent lung cancer (15). Considering the high incidence of infectious pulmonary events occurring in patients treated with pralsetinib, confusion between pulmonary progression and infectious adverse events might occur, as it was the case for our patient, and must be taken into account.

In the case of our patient, it is also important to note that the continuation of pralsetinib, even at lower doses, is associated with the onset of new infectious events (7, 9, 12).

A rapid clinical and imaging response followed by multisite relapse after the discontinuation of pralsetinib, but with an objective response at reintroduction, confirms the nature of a *RET* oncologic tumor in our patient. This exposure–response relationship was also observed in patients with *RET* fusion thyroid cancer treated with pralsetinib, in which patients with the highest plasmatic concentrations had the longest PFS duration, but this benefit was not observed in patients with NSCLC (16). Patients who initiated pralsetinib at 400 mg as opposed to 300 mg or less—both in NSCLC or thyroid cancers—also experienced the longest progression‐free survival (PFS) duration. The same study also reported a significantly increased risk of developing grade III or higher pneumonia and lymphopenia due to increased exposure to pralsetinib (16). This observation is partially consistent with the history of our patient, in whom most opportunistic infections occurred while receiving 400 mg/day of pralsetinib. However, after an infectious adverse event, the timing and resuming dose of pralsetinib remain unknown and dependent on local expertise.

**Figure 2:**
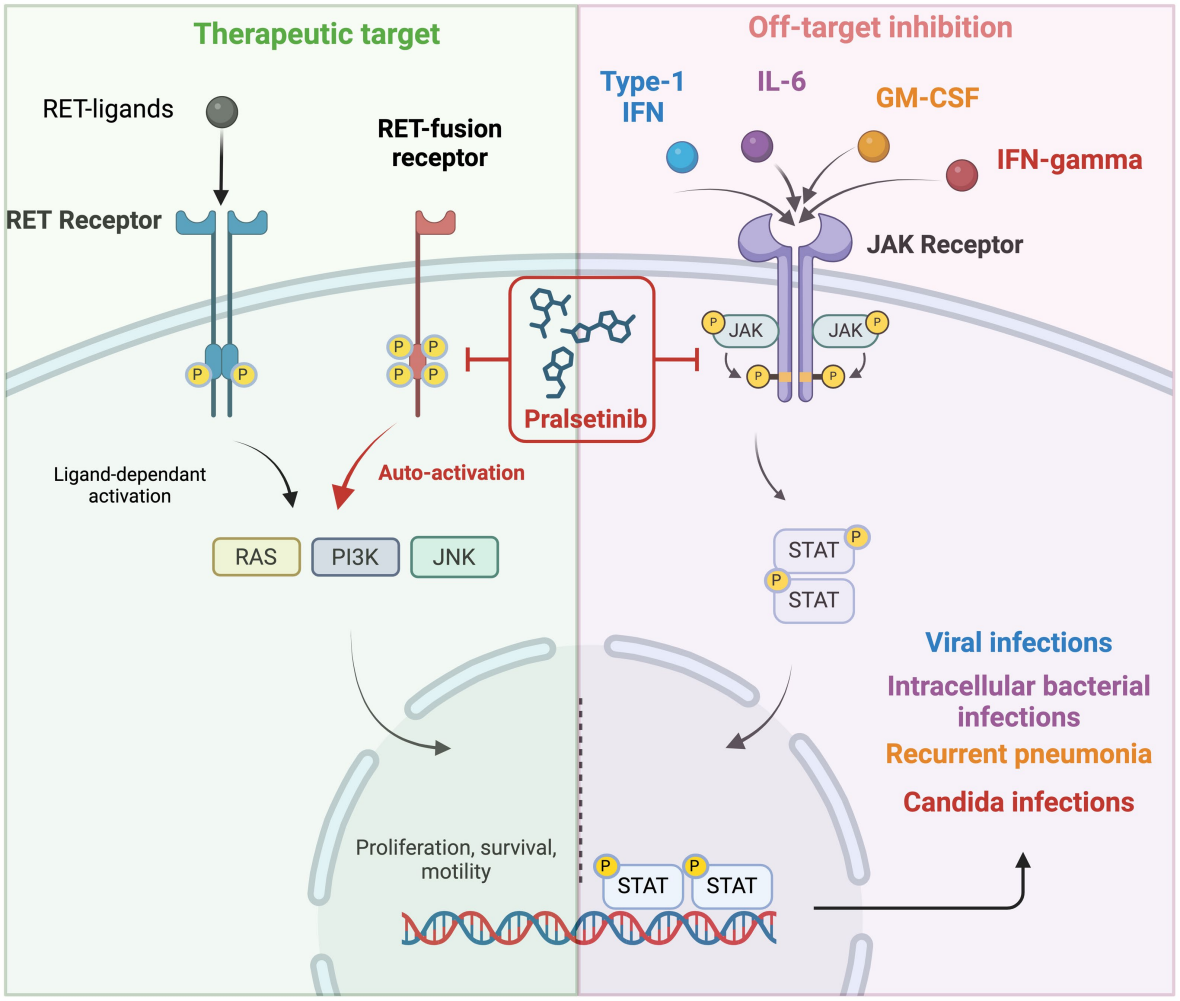
Pralsetinib off-target effect on JAK1/2 and infectious adverse events. The left panel is represented the pralsetinib therapeutic pathway. The right panel is presented pralsetinib’s off-target effect on the JAK/STAT signaling pathway. The common infections associated with JAK/STAT inhibition are viral infections, intracellular bacterial infections, recurrent pneumonia, and candida infections. We hypothesize that this off-target inhibition is involved in recurrent infectious events under pralsetinib treatment.

Considering these numerous infectious events, we first hypothesized the possibility of a class effect common to all RET inhibitors. However, no severe infections were reported with selpercatinib in clinical trials nor found in the French Pharmacovigilance Registry that we consulted in February 2024. It seems therefore unlikely that the high incidence of infectious events observed with pralsetinib is linked to a class effect of RET inhibitors. Our next hypothesis was an association with an off-target effect of pralsetinib. Indeed, while selpercatinib and pralsetinib share common off-target effects on VEGF1-3 and FGFR1-3 (5), selpercatinib specifically inhibits aurora kinase B, while pralsetinib has specific off-target inhibition on JAK1/2, DDR1, FLT3, PDGFRb, TRKA, and TRKC (5, 17). Among them, the JAK-mediated intracellular signaling pathways play a crucial role in immunoregulation and host defense. It is well established that for autoimmune diseases or cancers, JAK inhibitors are associated with an increased frequency of infection (18–20). Pralsetinib may therefore predispose patients to infections as a result of their off-target effects on JAK1/2. *In vitro*, pralsetinib has proven to have a highly specific inhibition on JAK1 and, to a lesser extent, JAK2, with a respective 16-fold and 136-fold shift increase in Ic_50_ as compared to RET Ic_50_ (17). *In vitro*, pralsetinib inhibition of JAK2 decreased STAT-5 phosphorylation (5). Physiologically, the RET pathway is intercrossed with the JAK/STAT pathway, and in addition to JAK1/2 off-target inhibition, RET inhibition by pralsetinib can lead to a decrease in JAK/STAT activation (21) (Figure 2). However, this pathway does not appear to be the most relevant, considering the low frequency of infectious events observed in other RET inhibitors. Considering the key role of the JAK/STAT pathway in innate immunity, immune tolerance (22), cytokine cascade, and interferon gamma response (23), we can hypothesize that a spectrum of pralsetinib infections might be related to the off-target inhibition of the JAK/STAT pathway.

Finally, there were no iatrogenic or confounding risk factors that could have interfered with the pralsetinib treatment. Notably, Charcot–Marie–Tooth disease affecting our patient is not associated with immunosuppression or recurrent infectious events.

## Conclusion

In addition to pneumonia, recurrent and serious infectious adverse events appear to be of concern under the pralsetinib therapy but not reported with selpercatinib. These infections might be wrongly interpreted as disease progression, despite the clinical activity of pralsetinib, and could be related to specific off-target inhibition of the JAK1/2 pathway. Early detection and timely application of anti-infective drugs are key to treatment. Further studies are needed to provide proper guidelines to physicians for managing infectious adverse events under pralsetinib therapy.

## Data availability statement

The raw data supporting the conclusions of this article will be made available by the authors, without undue reservation.

## Ethics statement

Written informed consent was obtained from the individual(s) for the publication of any potentially identifiable images or data included in this article.

## Author contributions

FP: Conceptualization, Investigation, Methodology, Supervision, Validation, Writing – original draft, Writing – review & editing. MJa: Investigation, Writing – review & editing. CG-R: Data curation, Investigation, Supervision, Writing – original draft. IK: Writing – review & editing. GL: Writing – review & editing. MJo: Writing – review & editing. JM: Writing – review & editing, Investigation. RG: Writing – review & editing. NF: Writing – review & editing, Investigation, Methodology, Supervision. EA: Investigation, Methodology, Supervision, Writing – review & editing, Conceptualization, Data curation, Formal analysis, Funding acquisition, Project administration, Resources, Software, Validation, Visualization, Writing – original draft.
